# Alamandine and Its Receptor MrgD Pair Up to Join the Protective Arm of the Renin-Angiotensin System

**DOI:** 10.3389/fmed.2019.00107

**Published:** 2019-06-11

**Authors:** Johanna Schleifenbaum

**Affiliations:** Institute of Vegetative Physiology, Berlin Institute of Health, Charité-Universitätsmedizin Berlin, Corporate Member of Freie Universität Berlin, Humboldt-Universität zu Berlin, Berlin, Germany

**Keywords:** alamandine, MrgD, GPCR, renin-angiotensin system, hypertension

## Abstract

Only a few years ago, alamandine was found to be a member of the protective arm of the renin-angiotensin system. It turned out to be an endogenous ligand of the G protein-coupled receptor MrgD. So far, MrgD had predominantly been studied in a neuronal context. The expression of the receptor in non-neuronal tissue showed hitherto unknown effects mediated by MrgD, most strikingly alamandine-induced vasodilation. Alamandine being a part of the non-classical renin-angiotensin system, a protective role of receptor activation seemed natural. This review summarizes the different effects of MrgD activation by alamandine in vasculature, in the central nervous system, and in organs as kidney and heart. Alamandine and MrgD are promising novel drug targets to protect the kidney and heart through anti-hypertensive actions.

## Introduction

**Figure 1:**
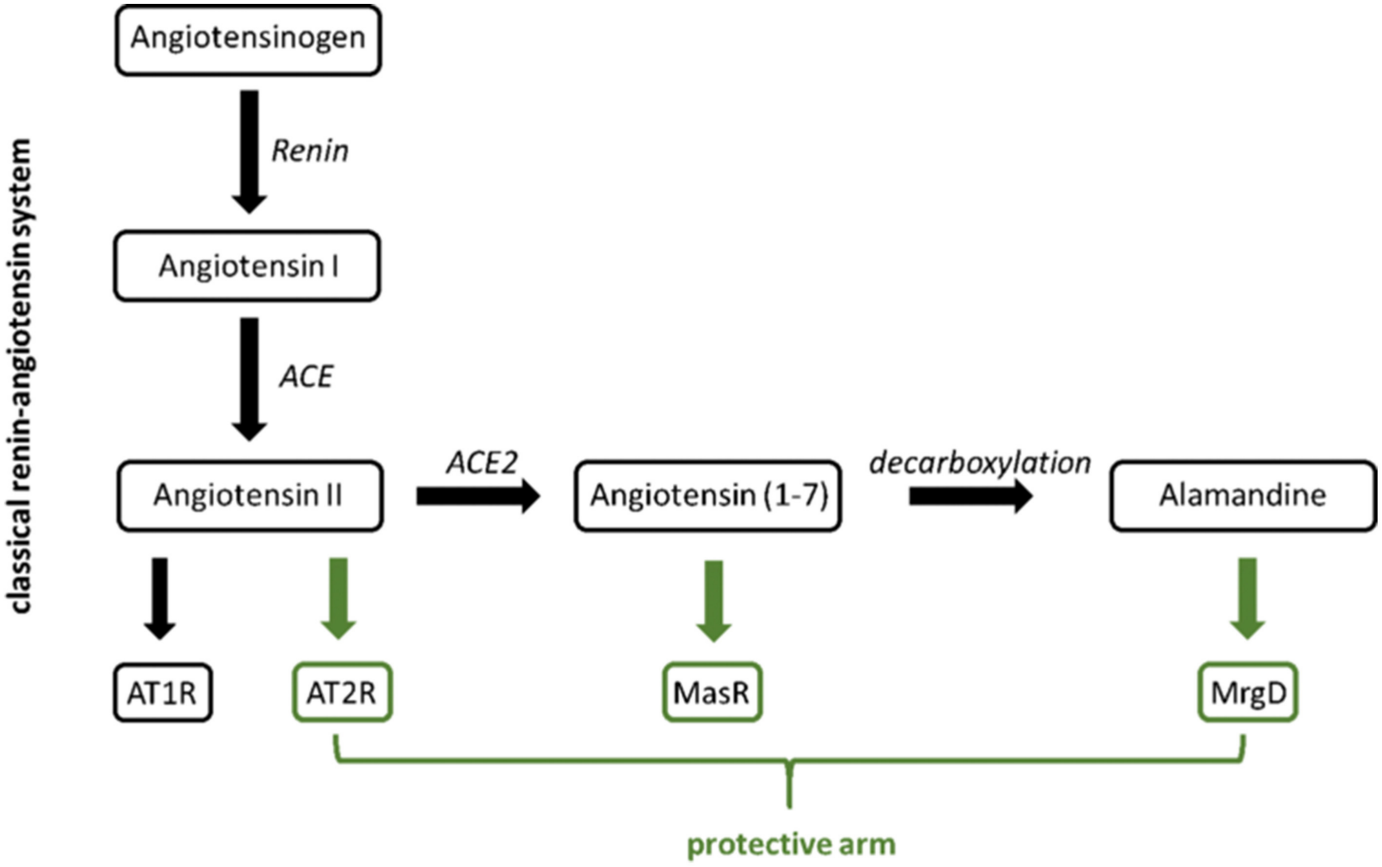
Classical (black) vs. protective arm (green) of the renin-angiotensin system.

When it comes to discussing G protein-coupled receptors (GPCRs) involvement in the development of hypertension, the first molecule that comes into everyone's mind is usually the angiotensin II receptor. The combination of the search terms “angiotensin receptor” and “hypertension” presents more than 7,000 publications on Pubmed. Many of those publications discuss the angiotensin II receptor subtype 1 (AT1R) as the “bad guy” promoting the development of hypertension, inflammation, remodeling, and more. AT1R inhibition reduces blood pressure and has anti-inflammatory and anti-proliferative effects. An increasing number of publications emphasize the role of the angiotensin II receptor subtype 2 (AT2R) in the so-called protective arm of the renin-angiotensin system (RAS). Following the development of hypertension research, one will soon come across another receptor belonging to the protective arm of the RAS: the Mas receptor. The search terms “Mas receptor” and “hypertension” still offer about 200 publications in Pubmed. The Mas receptor can be activated by its ligand angiotensin (1-7) [Ang (1-7)]. Derived from angiotensin II (Ang II) by Ang II-converting enzyme 2 (ACE2), it shows vasodilating (thus protective) properties. Ang (1-7) can be decarboxylated to a peptide called alamandine. Interestingly, alamandine was found to be an endogenous ligand for a GPCR related to Mas, the Mas-related G protein-coupled receptor member D (MrgD), also known as TGR7 (1) or hGPCR45 (2). The structural resemblance of the ligands Ang (1-7) and alamandine as well as of the receptors Mas and MrgD (3) suggests a possible role of MrgD in blood pressure regulation and hypertension, respectively (Figure 1). Very surprisingly, the search term “MrgD” provides only 42 publications in total on Pubmed. This can be explained by the fact that the investigation of the MrgD receptor in non-neuronal tissue started only a few years ago.

## What is Known About the MrgD Receptor?

The responsible gene *Mrgprd* is found in rodents as well as in humans (4), the coding sequence is located within a single exon (5). First publications described the receptor expression as constrained to a subset of pain-sensitive small-diameter neurons (1, 4–6). The MrgD receptor was associated with a role in the modulation of neuropathic pain (4, 5) and the perception of itching (7). Coexpression with MrgE, another member of the Mrg family, was found in macaque peripheral nociceptive neurons (8); both subtypes were shown to form heteromeres in HEK cells (9). Expression of several Mrg receptor subtypes was increased in mouse inflamed intestinal tissue (10, 11).

In a cardiovascular context, expression was detected in arterial smooth muscle cells, endothelial nitric oxide synthase (eNOS)-positive endothelial cells, and in atherosclerotic plaques (12). Oliveira et al. localized the receptor in blood vessels, cardiomyocytes (mainly in the membrane, perinuclear, and nuclear region), and the cardiovascular center of the mouse brain. They also studied MrgD-deficient mice and found left ventricular remodeling and a pronounced dilated cardiomyopathy, decreasing the systolic function of the mice (13). If treated with angiotensin II, hearts of spontaneously hypertensive rats (SHR) and cardiomyocytes showed an increased expression of MrgD. The MrgD ligand alamandine was able to attenuate hypertension and alleviate cardiac hypertrophy in this model (14).

## G Protein Coupling

The MrgD receptor is G protein-coupled. Most studies were performed in heterologous expression systems, interestingly suggesting a possible coupling to different heterotrimeric G protein subtypes (Table 1). Shinohara et al. were the first ones to show that ß-alanine, a neurotransmitter and a ligand of the MrgD receptor, initiates calcium influx into MrgD-expressing Chinese hamster ovary (CHO) cells (indicating G_q_ protein coupling), but also reduces forskolin-induced cAMP production (sensitive to pertussis toxin, thus indicating G_i_ protein coupling) (6). The ß-alanine-induced receptor activation also increased intracellular calcium concentration and stimulated ERK1/2 phosphorylation in Human embryonic kidney 293 (HEK293) cells (9). In cells with coexpression of MrgD and MrgE, ß-alanine-induced ERK1/2 phosphorylation was increased, while MrgD internalization was reduced followed by a prolonged calcium influx (9). Coexpression of MrgD and voltage-sensitive KCNQ2/3 potassium channels in HEK293 cells resulted in a strong inhibition of KCNQ2/3 currents (neuronal “M current”) upon ß-alanine-induced receptor activation. This effect was blocked completely by phospholipase C (PLC) inhibition (again indicating G_q_ protein coupling) and partially by pertussis toxin (indicating G_i_ protein coupling). Interestingly, results were partially confirmed when experiments have been performed in isolated DRG neurons. Here, KCNQ2/3 current activation was partially inhibited by PLC blockade and blocked completely by pertussis toxin (15).

**Table 1:** Overview of MrgD receptor G protein-coupling under different conditions.

**G protein**	**Effect**	**Expression system or tissue**	**Ligand**	**References**
G_q_	Ca^2+^ influx	CHO cells + MrgD expression	ß-alanine	(6)
G_q_	Ca^2+^ influx prolonged Ca^2+^ influx	HEK293 cells + MrgD expression + MrgD, MrgE coexpression	ß-alanine	(9)
G_q_	Inhibition of KCNQ2/3 current	CHO cells + MrgD, KCNQ2/3 coexpression	ß-alanine	(15)
G_q_	Partial inhibition of KCNQ2/3 current	Isolated DRG neurons	ß-alanine	(15)
G_q_	Opening of CACC channels	oocytes + MrgD expression	ß-alanine	(16)
G_q_	Decreased secretion, expression, and blood level of leptin	Perirenal adipose tissue	alamandine	(17)
G_s_	Increased cAMP level	HEK293 cells + MrgD expression	Ang (1-7)	(18)
G_s_	Increased cAMP level	Mesangial cells	Ang (1–7)	(18)
G_s_(G_i_)	Increased cAMP level	Primary mesangial and endothelial cells	Alamandine (higher doses)	(19)
G_i_	Decreased cAMP level	CHO cells + MrgD expression	ß-alanine	(6)
G_i_	Partial inhibition of KCNQ2/3 current	CHO cells + MrgD,KCNQ2/3 coexpression	ß-alanine	(15)
G_i_	Inhibition of KCNQ2/3 current	Isolated DRG neurons	ß-alanine	(15)
G_i_	Reversal of hyperhomocysteinamia-induced vascular dysfunction	Isolated abdominal aorta of New Zealand White rabbits	alamandine	(20)
G_i_	Increase in blood pressure and sympathetic outflow	Microinjection in paraventricular nucleus of WKY and SHR rats	alamandine	(21)

While opening of KCNQ channels hyperpolarizes the cell membrane, opening calcium-activated chloride channels leads to a depolarization. KCNQ channels can be inhibited by PIP_2_ depletion, thus by activation of G_q_ protein-coupled receptors. An increase in intracellular calcium, also resulting from G_q_ protein activation, opens calcium-activated chloride channels. Thus, it was not very surprising that in MrgD-expressing oocytes, a ß-alanine-induced current was found to be sensitive to calcium-activated chloride channel inhibitors and calcium chelators. The chloride current was reduced by phospholipase C (PLC) inhibition and by inositol 1,4,5-trisphosphate (IP_3_) receptor antisense nucleotides, while phosphokinase C (PKC) inhibition had no effect (16). This indicates a signal transduction from MrgD to CaCC via calcium as second messenger, while the transduction pathway diacylglycerol (DAG)/PKC is neglectable under those conditions.

Thus, ß-alanine is able to signal via MrgD receptor activation inducing G_q_ or G_i_ protein-coupled pathways. It remains unclear, which transduction is used under physiological conditions, though both pathways are involved in the regulation of neuronal excitability. This suggests a limitation of ß-alanine as a ligand of MrgD receptors expressed in neurons.

Other ligands of the MrgD receptor are more relevant in the cardiovascular context. Ang (1-7), known as a ligand for the Mas receptor, is also binding to the MrgD receptor and stimulating the G_s_ protein-coupled signaling pathway. Moreover, it increased cyclic adenosine monophosphate (cAMP) concentrations in MrgD-transfected HEK293 cells and in mesangial cells, but not in MrgD-deficient mesangial cells (18). Similar effects were found for alamandine, an endogenous MrgD ligand circulating in the human blood. It elevated cAMP concentration in primary endothelial and mesangial cells (18), also suggesting G_s_ coupling. Interestingly, higher concentrations of alamandine were also involved in the G_i_-coupled pathway in primary mesangial and endothelial cells, showing specific pharmacodynamics properties of alamandine compared to Ang (1-7) (19). Reversal of hyperhomocysteinamia-induced vascular dysfunction by alamandine in isolated abdominal aorta of New Zealand White rabbits was dependent on G_i_ protein signaling (20). In blood vessels of New Zealand White rabbits on an atherogenic diet and control animals, alamandine enhanced acetylcholine-mediated vasodilation in thoracic aorta, and iliac artery, while vasodilation of renal arteries by acetylcholine was reduced. In total, the vasoreactivity of alamandine was reduced in atherogenic arteries compared to controls (12). *In vivo*, participation of the G_i_ signaling pathway in an alamandine effect could be shown after microinjection of alamandine in the paraventricular nucleus of normotensive Wistar Kyoto (WKY) rats and SHR rats. Blood pressure as well as sympathetic outflow increased in both groups upon activation of MrgD receptor dependent on cAMP and protein kinase A (PKA), while the increase in SHR rats was larger than in the control group (21).

Alamandine decreases secretion, expression, and blood levels of leptin. When leptin levels are low, expenditure is reduced and hunger is increased (22). Genetic deficiency of leptin or its receptor, in both mice and humans, “fools” the brain into thinking that fat stores are absent, resulting in extreme hunger and obesity (22). However, the role of leptin in maintaining energy balance is asymmetric; low levels strongly promote restoration of fat stores, whereas high levels weakly resist obesity. Another feedback signal probably serves this latter function (i.e., to resist obesity), but the identity of the signal (or signals) is unknown (22). Remarkably, leptin plasma levels are elevated under hypertensive conditions (23). This suggests a potentially protective role of the protein. This effect in perirenal adipose tissue of Wistar rats was mediated by G_q_ protein signaling and also involved c-Src, p38 mitogen-activated protein, and IκB activation (17). Additionally, alamandine induced expression of iNOS and plasminogen activator inhibitor-1 (PAI-1) in adipose tissue and isolated adipocytes (17).

## Alamandine and Vasodilation

Reduction in leptin expression by alamandine was reversed by the NOS inhibitor L-NG-nitroarginine methyl ester (L-NAME), while leptin expression was inhibited by S-nitroso-L-glutathione, an donor of nitric oxide (NO) (17). The vasodilating molecule NO is produced by the enzymes iNOS, eNOS, and nNOS. Indeed, observed vasoreactive effects of alamandine are mediated by NO release. Leao et al. compared the vasorelaxing effects of alamandine in spontaneously hypertensive stroke prone (SHRSP) rats. They found an enhanced vasorelaxation in SHRSP rats mediated by release of NO and prostaglandins^1^. At least in aortic rings, the vasodilating effects of alamandine are sensitive to inhibition of endothelial NO release by L-NAME. Also, they can be blocked by ß-alanine, probably through competitive inhibition (24).

The literature about vasodilating effects of alamandine mediated through MrgD receptor activation is very rare; some studies are available only as abstracts. Local vasodilation, particularly in arterial beds with high peripheral resistance, could be a powerful tool in fighting hypertension. Thus, further studies are needed to investigate the involvement of the MrgD receptor and its ligand alamandine. Both molecules, i.e., MrgD and alamandine, could be of interest for the development of new therapies against cardiovascular diseases. The fact that the new RAS hormone alamandine can be easily administered orally as a HPβCD inclusion compound and produced antihypertensive effects in spontaneously hypertensive rats and pronounced protective effects in cardiac fibrosis, opens new perspectives for exploring the therapeutic potential of angiotensin-(1–7)–related peptides. The identification of 2 novel components of the RAS, alamandine and its receptor, will be important for improving our understanding of the physiological and pathophysiological role of the this key regulatory system. It is possible that these 2 novel compounds of the RAS play a pathophysiological role in kidney failure since hemodialysis patients seem to exhibit increased serum concentrations of alamandine (as measured by alamandine /Ang II ratios in 5 patients) (24).

## MrgD Receptors, Alamandine, and Cardiovascular Function

Several studies have been published showing an effect of alamandine-induced MrgD activation on blood pressure. In renovascular hypertensive rats, alamandine showed a biphasic hemodynamic effect after infusion: AT1R-mediated signaling by alamandine under normal conditions (normotensive rats) is superimposed by activation of a PD123319-sensitive receptor under pathophysiological conditions (renovascular hypertensive 2K1C rats) (25). PD123319 has been regarded as an specific AT2R inhibitor, but also inhibits MrgD receptors, as found in experiments using knock-out animals (26). This study is the only one hypothesizing so far that alamandine is also signaling via AT1R activation. The authors suggest an agonistic effect of alamandine based on the knowledge of the structural related Ang (1-7) being an AT1R agonist (27). When injected into the caudal ventrolateral medulla, alamandine showed an MrgD-dependent hypotensive effect in normotensive control rats, while the blood pressure curve was U-shaped in 2K1C animals (28). *In vivo*, oral doses of alamandine lead to a long-lasting antihypertensive effect in SHR rats, as well as to an anti-fibrotic affect in rats treated with isoprotenerol (24). Effects specifically in the heart were seen upon treatment of cultured neonatal rat cardiomyocytes with alamandine. The treatment prevented angiotensin II-induced hypertrophy via MrgD activation involving a 5′ AMP-activated protein kinase (AMPK)/NO pathway (29). In the same cell type, and additionally in mice treated with lipopolysaccharide (LPS), alamandine attenuated sepsis-induced cardiac dysfunction via inhibition of mitogen-activated protein kinase (MAPK) signaling pathways. Alamandine prevented myocardial inflammation, apopotosis, and autophagy induced by lipopolysaccharides (LPS). It also reversed decreases in cardiac ejection fraction and NOS expression, leading to reduced blood pressure (30). As previously mentioned, administration of alamandine attenuated hypertension and alleviated cardiac hypertrophy in angiotensin II-treated SHR hearts (14). In normotensive SD rats, alamandine administration *in vivo* increased the plasma level of atrial natriuretic peptide (ANP) via MrgD activation. It was also improving postischemic left ventricular pressure and decreasing the infarct size, while decreasing apoptotic protein and increasing antioxidative protein expression (31).

## Summary

There are only a few studies about the MrgD receptor and its endogenous ligand alamandine, but many of them show a clear relevance of both molecules for the cardiovascular system. Though the molecular details of the signaling pathways are not completely evolved yet, some physiological effects of receptor and ligand are proven.

Most striking is an increased expression of NOS enzymes upon alamandine-induced activation of MrgD, leading to NO-mediated vasodilation. The induced vasodilation is sufficient for a reversal of vascular endothelial dysfunction as well as an alleviation of the venous return to the heart, thus decreasing the preload of the heart. The vasodilation is subsidized by an alamandine-induced increase in ANP plasma levels. The depressor effect of MrgD activation is masking a preceding pressure effect in healthy animals, while the mechanism seems dysfunctional in rat models of hypertension. Also relevant is an anti-fibrotic effect, preventing hypertrophy of cardiomyocytes. The protective impact of alamandine-induced MrgD activation is strengthened by changes in expression of proteins improving the outcome of cardiac infarcts.

Though studies investigating alamandine and/or MrgD receptor are done in different models, the findings are consistent and fit into a bigger picture. All alamandine-induced MrgD receptor actions are affecting the cardiovascular system, so targeting both molecules can be a worthwhile goal for the development of future antihypertensive and cardioprotective drugs.

## Author Contributions

JS sifted all publications regarding the topic, drafted, and finalized the manuscript.

### Conflict of Interest Statement

The author declares that the research was conducted in the absence of any commercial or financial relationships that could be construed as a potential conflict of interest. The handling Editor declared a shared affiliation, though no other collaboration on this topic or techniques, with the author.

## Footnotes

^1^https://www.ahajournals.org/doi/10.1161/hyp.68.suppl_1.p281
